# Present and Emerging Biomarkers in Immunotherapy for Metastatic Non-Small Cell Lung Cancer: A Review

**DOI:** 10.3390/curroncol29020043

**Published:** 2022-01-21

**Authors:** Raida M. Khwaja, Quincy S.-C. Chu

**Affiliations:** Division of Medical Oncology, Department of Oncology, Cross Cancer Institute, University of Alberta, Edmonton, AB T6G 1Z2, Canada; Raida.khwaja@albertahealthservices.ca

**Keywords:** PD(L)1, immunotherapy, predictive biomarkers, non-small cell lung cancer

## Abstract

Targeting the immune system, especially the PDL-1/PD-1 axis, has significantly improved the outcomes of metastatic lung cancer patients. However, only a portion of patients will benefit significantly from PD(L)1 therapeutics alone or in combination with either chemotherapy or anti-CTLA4 antibody. It is therefore important to study predictive biomarkers to help select the patients who will experience the most benefit from immunotherapy. In this paper, the current status of PDL-1 expression on tumour cells, the smoking status of patients, tumour mutational burden, gut microbiome and STK11 and KEAP1 mutations in the tumour as predictive biomarkers for PD(L)-1-based immunotherapy are summarized.

## 1. Introduction

Cancer immune surveillance’ refers to the theory that the immune system plays a protective role by recognizing cancer cells, inhibiting their growth and inducing apoptosis. The ability to evade this protective mechanism has become an important and established hallmark of cancer [1]. The development of immunotherapy, which manipulates the immune system to overcome this proposed cancer growth mechanism, has changed the landscape of prognosis of metastatic non-small cell lung cancer (mNSCLC). Prior to the era of immunotherapy, the standard first-line treatment for metastatic NSCLC, with no actionable mutations, was doublet platinum-based chemotherapy. Several studies have now shown increased benefit with immunotherapy in the metastatic setting. However, while many patients benefit from immunotherapy, there are others who do not. Therefore, there have been ongoing studies to determine what predictive factors will determine which patients would benefit from immunotherapy.

Predictive biomarkers are characteristics that affect how effective a particular treatment will be in a given patient population. This article reviews the role of PD-L1 expression, tumour mutational burden (TMB), smoking history, STK11/KEAP1 mutations and the gut microbiome as predictive markers for immunotherapy. The role of these factors in immunotherapy benefit for metastatic NSCLC, which is defined by overall response and median overall survival, will be discussed.

## 2. The Role of PD-L1 as Predictive Biomarker

**Figure 1:**
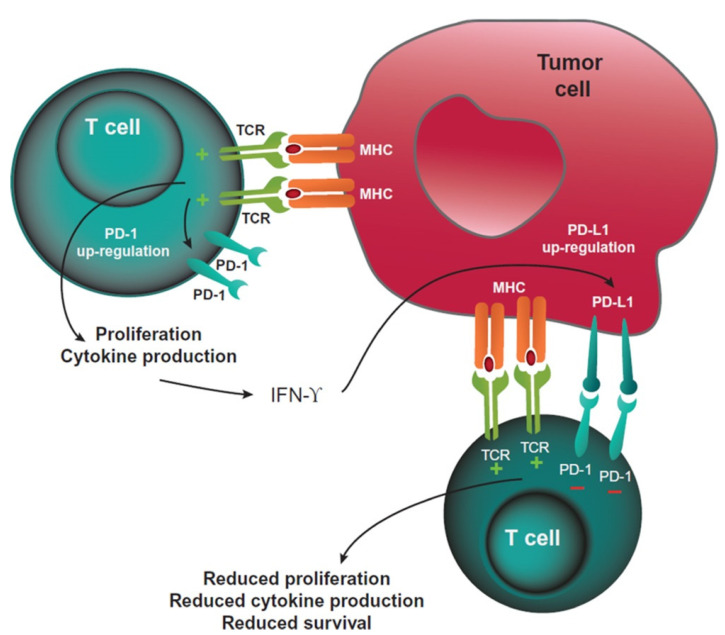
PD-1-mediated inhibition of T cells. Reprinted from Ref. [3].

The cytotoxic T-cell response can be activated by the stimulation of agonistic immune checkpoints, such as OX40 and GITR, or inhibited by antagonistic checkpoints, such as CTL Antigen-4 (CTLA-4) and Programmed Death 1 (PD-1, Figure 1), simultaneously [2,3]. CTLA-4 is a protein located in the membrane of T cells that controls T-cell clonal selection, proliferation, maturation, and the early stages of T-cell activation. PD-1 is a membrane protein that is also located on the surface of T cells, involved in the later stages of T-cell activation. When PD-1 binds to its ligands B7-H1/PD-L1 located on tumour cells, it inhibits the activity of the T cell. These proteins that inhibit the response are known as “checkpoint inhibitors”. B7-H1 is a co-inhibitor with PD-L1. When both molecules bind to the ligand, PD-1 of T lymphocytes inhibits the T-cell function, leading to T-cell exhaustion and immune evasion. Immunotherapy blocks this receptor–ligand interaction and allows the immune system to be activated and control the cancer cells’ proliferation and metastases.

Nivolumab is a PD-1 inhibitor and has been shown to improve overall survival compared with single-agent docetaxel in the second-line setting for squamous cell NSCLC [4]. This phase 3 clinical trial included patients with metastatic squamous cell NSCLC who progressed on platinum doublet chemotherapy. The median overall survival (mOS) was 9.2 months with nivolumab vs. 6.0 months with docetaxel (hazard ratio (HR) = 0.59, 0.44–0.79, *p* < 0.001). The tumour tissues were assessed for the quantification of PD-L1 expression, with prespecified expression levels of 1%, 5%, or 10%. The objective response rates (ORR) to nivolumab were similar regardless of the PD-L1 expression. Examining the median mOS for nivolumab-treated patients by PD-L1 expression, the interaction *p*-values for >1%, >5% and >10% PD-L1 expression were 0.556, 0.4747 and 0.4062, respectively. The mOS for PD-L1 expression <1% vs. >10% was 8.7 months vs. 11 months, respectively. Overall, the mOS was consistently better in the nivolumab group regardless of the PD-L1 expression level, when compared with chemotherapy. This suggested that the PD-L1 expression may not be a prognostic or predictive marker. However, the study also suggested that one of the reasons for this finding may be attributed to the fact that only 83% of the patient samples were available for the quantification of PD-L1 expression, and the samples were archival. Both factors may have affected the quality of the assay, thereby not reflecting the PDL-1 expression after prior systemic therapy.

In the second-line setting, nivolumab was also shown to improve survival in previously treated non-squamous NSCLC patients, compared with docetaxel [5]. The mOS with nivolumab was 12.2 months vs. 9.4 months with docetaxel ((HR = 0.73, 96% confidence interval (CI) 0.59–0.89, *p* = 0.002). The ORR to nivolumab for those patients with PD-L1 expression <1%, 1–5%, 5–10% and >10% was 9%, 31%, 36% and 37%, respectively. The mOS for PD-L1 expression <1% vs. >10% was 10.5 months vs. 19.4 months, respectively, suggesting a greater magnitude of benefit to nivolumab in those patients who express PD-L1 than those who do not. This was the first clinical trial to show a predictive association between PD-L1 expression level and benefit of immunotherapy.

Other trials demonstrating an association between PD-L1 expression with median progression-free survival (mPFS) and mOS were the KEYNOTE-010 trial and the OAK trial (6,7). In the KEYNOTE-010 trial, patients with at least 1% PDL-1 expression were randomized to pembrolizumab 2 mg/kg, 10 mg/kg, or docetaxel 75 mg/m^2^ every 3 weeks [6]. The subgroup analysis of mPFS also showed improvement in the pembrolizumab arm for both TPS 1–49% and >50%. However, the HR was not statistically significant for TPS 1–49% with HR for 1.04 (95% CI 0.85–1.27). In comparison, for PD-L1 TPS of >50%, the HR was 0.59 (95% CI 0.46–0.74). In terms of mOS, the PD-L1 expressions of 1–49% and >50% both had statistically significant improvements in mOS, of 10.4 months and 12.7 months, respectively, compared with docetaxel, of 8.5 months. The OAK trial investigated docetaxel vs. atezolizumab, an IgG1 monoclonal antibody that targets PD-L1/PD-1 and PD-1/B7-H1 interactions in those who progressed on at least platinum-based chemotherapy [7]. The mOS HR was 0.75 (95% CI 0.59–0.96) in patients who received atezolizumab with PDL-1 expression of less than 1% tumour cells or tumour-infiltrating immune cells. The greatest benefit was derived in patients with high PD-L1 in TC3 and IC3 (TC3 defined as PD-L1 expression on >50% of tumour cells and IC3 defined as >10% or more of tumour-infiltrating immune cells), with a HR for mOS of 0.41 (95% CI 0.27–0.64). Moreover, the ORR with PD-L1 expression of >50% and PD-L1 <1% expression was 31% vs. 8%, respectively, in the atezolizumab arm. Together, these data demonstrate that the greater the PD-L1 expression, the higher the benefit is in terms of mOS and ORR with atezolizumab.

In the first-line setting of metastatic NSCLC, the KEYNOTE-024 trial was the first phase 3 clinical trial that demonstrated an improvement in mOS in patients whose tumour expressed PD-L1 >50% [8]. The HR for mOS was 0.49 (95% CI 0.34–0.69), favouring pembrolizumab over platinum-based chemotherapy. Additionally, in the KEYNOTE-042 trial, patients with PD-L1 of 50% or greater had benefit in terms of overall survival compared with patients with PD-L1 of 1–49% [9]. The HR for mOS was 0.69 (95% CI 0.56–0.85, *p* = 0.0003) and 0.92 (95% CI 0.77–1.11, *p* = 0.0020) for PD-L1 >50% and 1–49%, respectively. In conclusion, these data also support the predictive role of PD-L1 expression in response to immunotherapy.

Gandhi et al. demonstrated that mNSCLC patients with adenocarcinoma who received four cycles of a platinum-based doublet chemotherapy with pembrolizumab, followed by pembrolizumab and pemetrexed, had improved mOS to platinum/pemetrexed, regardless of the PDL-1 expression level [10]. The HR for mOS for PD-L1 <1%, 1–49% and >50% was 0.59 (95% CI 0.38–0.92), 0.55 (95% CI 0.34–0.90) and 0.42 (95% CI 0.26–0.68), respectively. The 12-month survival rate in patients with PDL-1 <1%, 1–49% and >50% was 61.7%, 71.5% and 73%, respectively. Although benefit was demonstrated in all PD-L1 subgroups, consistent with other studies, those with PD-L1 >50% derived the highest benefit from pembrolizumab.

In contrast, KEYNOTE-407 reported that patients with metastatic squamous cell NSCLC who received chemotherapy in combination with pembrolizumab demonstrated comparable mOS benefit, with PDL-1 <1%, 1–49% and >50% resulting in HR of 0.61 (95% CI 0.38–0.98), 0.57 (95% CI 0.36–0.90) and 0.64 (95% CI 0.37–1.10), respectively [11]. This represents the difference in the behaviour of squamous cell carcinoma compared with non-squamous cell NSCLC.

In the IMpower130 study, patients with advanced metastatic NSCLC non-squamous cell subtype were randomized to platinum-based chemotherapy vs. atezolizumab with chemotherapy [12]. The HR for mOS favoured the immunotherapy/chemotherapy arm. The HR for mOS for PD-L1 high vs. PD-L1 negative were 0.84 (95% CI 0.51–1.39) vs. 0.81 (95% CI 0.61–1.08), favouring the experimental arm, although the CI crossed 1. While this is not statistically significant, there was still clinical benefit present in the immunotherapy arm, regardless of PD-L1 status.

The IMpower150 study was another phase 3 clinical trial that evaluated atezolizumab plus carboplatin plus paclitaxel (ACP), bevacizumab with carboplatin plus paclitaxel (BCP) and atezolizumab plus BCP followed by maintenance therapy with atezolizumab. The addition of either bevacizumab or both bevacizumab and atezolizumab to chemotherapy in metastatic non-squamous NSCLC in the first-line setting improved the mPFS and mOS [13]. Furthermore, the HR for mPFS in the ABCP group vs. BCP group was 0.62 (95% CI 0.52–0.74, *p* < 0.001), while the HR for mOS of the ABCP vs. BCP group was 0.78 (95% CI 0.64–0.96, *p* = 0.02).

Reck et al. reported the 2-year update of Checkmate 9LA, which showed an improvement in mOS in patients with mNSCLC treated with a combination of platinum-based chemotherapy and immunotherapy compared with platinum-based chemotherapy alone. The improvement occurred regardless of histology and PDL-1 expression (15.8 months vs. 11.0 months, HR = 0.72, 95% CI 0.61–0.86) [14]. The mOS benefit of two cycles of platinum-based chemotherapy in combination with nivolumab and ipilimumab was comparable regardless of PDL-1 status (HR = 0.67, HR = 0.70 and HR = 0.67 for PDL-1 <1%, PDL-1 1–49% and PDL-1 >50%, respectively).

In summary, in both the first- and second-line setting, PDL-1 expression is a predictive biomarker for therapeutic response to single-agent immunotherapy, mPFS and mOS (Table 1). Additionally, when immunotherapy is administered with chemotherapy, it was also demonstrated to be a biomarker for clinical outcome. However, it is also evident that metastatic squamous cell NSCLC patients tend to have greater benefit with immunotherapy regardless of the PD-L1 status. This indicates that there may be other predictive markers that influence benefit to immunotherapy.

## 3. Smoking History

Smoking remains one of the most important risk factors for lung cancer, accounting for 80%–90% of lung cancer cases. Smoking history has also been studied as a predictive biomarker for benefit to immunotherapy. Cigarette smoking exposes an individual to carcinogens, which, in turn, can result in DNA mutations. It has been hypothesized that smoking results in the accumulation of mutations or neoantigens that allow the immune system to recognize them.

In their retrospective analysis, Nagahashi used next-generation sequencing of NSCLC tissue collected from 100 patients [15]. A high TMB, defined as >20 mutations per mega base (Mb), was found in 10% of the total patient population. Eighty percent of these patients had smoking history, whereas, in those with low TMB, 19% of the patients were current smokers (*p* < 0.001).

Several studies showed that the efficacy measured by overall ORR to immunotherapy is higher if they are current or former smokers. A study by Gainor [16] demonstrated, the ORR to PD-1/PDL-1 inhibitors was 4.2% and 20.6% among non-smokers and smokers, respectively [16]. Of note, non-smokers, who had a lower response to PD-1/PD-L1 inhibitors, were more likely to harbour EGFR mutation or anaplastic lymphoma kinase (ALK) rearrangements [5,6,7,17].

The subgroup analysis of KEYNOTE-024 showed that the mOS with pembrolizumab was higher than chemotherapy for current smokers and former smokers with an HR of 0.81 (95% CI 0.41–1.60) and 0.59 (95% CI 0.41–0.85), respectively [8]. In KEYNOTE-042 trial, the subgroup analysis showed that in the population of patients with PD-L1 >50%, non-smokers had an HR of 1.10 (95% CI 0.69–1.75), while former and current smokers had an HR of 0.60 (95% CI 0.46–0.80) and 0.71 (95% CI 0.43–1.16), respectively. This trend was observed across all PD-L1 subgroups [9]. However, in the IMpower132 study, the mOS HR for non-smokers was 0.78 (95% CI 0.42–1.43) while for current or former smokers it was 0.89 (95% CI 0.72–1.09) [18]. In IMpower130, the HR for mOS in non-smokers and current or previous smokers was 0.55 (95% CI 0.26–1.19) and 0.81 (95% CI 0.65–1.02), respectively [12].

In a retrospective analysis of 71 lung adenocarcinoma patients, individuals with current or past smoking history had a higher incidence of PD-L1 >50% expression (*p* = 0.0111) [17]. Pan et al. found a similar association in both squamous cell carcinomas and adenocarcinomas. In the adenocarcinoma subgroup, 93.8% of the patients who had PDL-1 TPS >50% were current or ex-smokers [19].

In CheckMate 017 by Borghaei et al., the non-smoking, previously treated, non-squamous NSCLC patients may derive a lesser survival benefit, where HR for non-smokers and former/current smokers was 1.02 (95% CI 0.64–1.61) and 0.70 (95% CI 0.56–0.86), respectively [5].

In the OAK trial, a phase 3 clinical trial by Rittmeyer et al., patients with squamous or non-squamous NSCLC who progressed on first-line chemotherapy were randomized to receive atezolizumab or docetaxel [7]. The mOS favoured atezolizumab in both smokers and non-smokers, where HR for mOS was 0.71 (95% CI 0.47–1.08) in non-smokers and 0.74 (95% CI 0.61–0.88) in current or previous smokers. Similarly, in the KN189 study, the combination of pembrolizumab and chemotherapy showed a similar mOS benefit in both smokers (HR = 0.54, 95% CI 0.41–0.71) and non-smokers (HR = 0.23, 95% CI 0.10–0.54).

In summary, it appears that while some studies showed that smokers have more benefit in mOS with immunotherapy, other studies showed a similar benefit between smokers and non-smokers. There is heterogeneity in the patient samples in that they vary amongst studies in terms of the definition of non-smokers vs. current or former smokers. Studies also do not specify what constitutes a current or former smoker in terms of packs of cigarettes per year smoked, which can certainly be a confounding factor. It is also known that smoking affects TMB, which could be the confounding factor affecting the benefit to immunotherapy. As such, smoking does not seem to be a strong predictive factor of benefit to immunotherapy.

## 4. Tumour Mutational Burden

**Figure 2:**
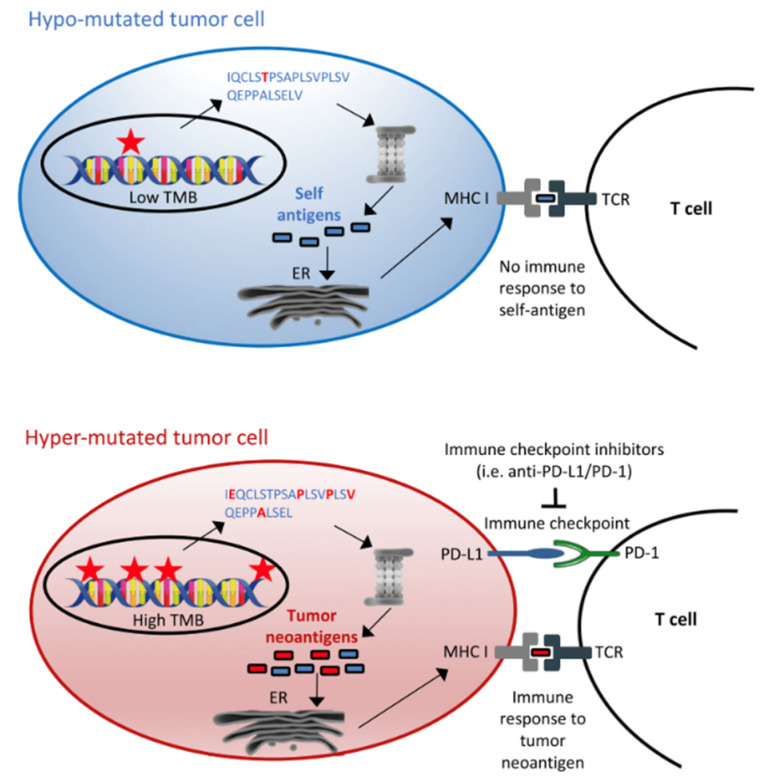
Tumour mutational burden as immunotherapy biomarker. Reprinted from Ref. [20].

TMB is defined as somatic mutations which include substitutions, insertions and deletions in any given gene (Figure 2) [20]. Individuals who are smokers tend to harbour greater TMB due to greater exposure to mutagenesis from carcinogens. These mutations result in the formation of neoantigens that are present on cancer cells, allowing the recognition and activation of the immune system. As a result, these individuals may be more likely to benefit from therapies such as immune checkpoint inhibitors (ICIs), which include PD-1/PDL-1 inhibitors. TMB (tumour mutational burden) can be measured in the tumour or the circulating tumour cell amount in the blood.

CheckMate 026 was a randomized trial which investigated a PD-1 inhibitor, nivolumab, and platinum-based chemotherapy in a first-line metastatic NSCLC setting without selection for PDL-1 status [21]. The mOS was in favour of chemotherapy for those with low or medium TMB with HR of 1.82 (95% CI 1.30–2.55). For high TMB, the HR for mOS was 0.62 (95% CI 0.38–1.00), favouring nivolumab. Most importantly, this study highlighted the importance of identifying predictive biomarkers in investigating benefit to immunotherapy. TMB became an even more important topic of exploration.

The CheckMate 227 trial was a phase 3 clinical trial investigating nivolumab and ipilimumab vs. nivolumab alone or doublet chemotherapy in mNSCLC with TMB >10 mutations per Mb [22]. In 58.2% of the patients whose tissue was evaluated for tumour TMB, the mOS was improved with immunotherapy regardless of the TMB. In a population with PD-L1 <1%, the HR for mOS for TMB <10 and >10 was 0.69 (95% CI 0.46–1.95) and 0.51 (95% CI 0.30–0.87), respectively. In a population with PD-L1 >1%, the HR for mOS for TMB <10 and >10 was 0.78 (95% CI 0.59–1.02) and 0.77 (95% CI 0.54–1.09), respectively. In a population with PD-L1 >50%, the HR for mOS for TMB <10 and >10 was 0.67 (95% CI 0.44–1.03) and 0.51 (95% CI 0.37–1.07), respectively. Numerically, nivolumab and ipilimumab offered survival benefit to patients with any PDL-1 or TMB status, but those with negative PD-L1 expression with TMB >10 may be the subgroup most likely to benefit from nivolumab and ipilimumab.

The MYSTIC study is a phase 3 clinical trial by Rizvi et al. investigating durvalumab with or without tremelimumab vs. standard doublet chemotherapy in first-line mNSCLC patients with PDL-1 >25% [23]. In the post hoc analysis, patients who had blood TMB (bTMB) >20 mutation per Mb, and had received the combination of chemotherapy and durvalumab/tremelimumab, displayed improved mOS with HR = 0.49 (95% CI 0.32–0.74) vs. patients with bTMB <20 mutation/MB, mOS with HR = 1.16 (95% CI 0.93–1.45). However, the bTMB of >20 mutations per Mb did not predict mOS benefit to chemotherapy and durvalumab, whereas tissue TMB (tTMB) > 10 mutation per Mb predicted the mOS benefit of both durvalumab alone, HR = 0.70 (95% CI 0.247–1.06), or durvalumab in combination with tremelimumab in addition to chemotherapy, HR = 0.72 (95% CI 0.48–1.09). This suggests that having a higher TMB may result in an improved outcome with immunotherapy, but whether blood or tumour TMB is a better predictive biomarker for durvalumab with or without tremelimumab and chemotherapy warranted further prospective investigation.

In the press release for the phase 3 NEPTUNE trial, the addition of tremelimumab with durvalumab to platinum-based chemotherapy in mNSCLC with bTMB > 20 mutation per Mb failed to show an improvement in mOS [24].

The TMB level also does not correlate with PD-L1 status, indicating that TMB is an independent predictive marker. Another important consideration in the future is the standardization of how tumour mutational burden is defined. The tTMB and bTMB have been shown to have positive correlation with mPFS and/or mOS; however, there remain technical differences in the sample collected that may account for the differences in results seen in their relationship with immunotherapy efficacy.

## 5. STK11, KEAP1

The serine/threonine kinase 11 (STK11) protein plays a role in the metabolism of lipids, glucose and cholesterol by activating the AMP-activated protein kinase [25]. Kelch-like ECH-associated protein (KEAP1) is an inhibitor of erythroid-related factor 2, which is involved in redox homeostasis, controlling multiple genes for detoxification and cytoprotective enzymes important for cellular stress from metabolic, oxidative stress, inflammation, and anticancer therapy [24]. Loss of function of this protein may allow cancer cells to proliferate and undergo metabolic reprogramming, and thus resist chemotherapy, radiotherapy, and immunotherapy. In human models and murine models, the inactivation of this protein results in reduced CD8^+^ T lymphocytes, which is indicative of a compromised tumour immune microenvironment. Several studies have proposed that one of the mechanisms of immune checkpoint inhibitor resistance is via mutations in STK11 and KEAP1 [25].

Papillon-Cavanagh et al. analysed the impact of STK11 and KEAP1 mutations in tumour samples from non-squamous NSCLC on the benefit of anti-PD-1/PDL-1 therapies, EGFR tyrosine kinase inhibitors, anti-vascular endothelial growth factor (VEGF), platinum-based combination chemotherapy or single-agent chemotherapy [26]. Amongst 2276 patients, mutations in STK11, KEAP1 and concurrent mutations in both STK11 and KEAP1 were detected in 20%, 20% and 10% of the total patient population, respectively. Furthermore, 75.8% of the STK11- and/or KEAP1-mutated samples had negative PD-L1 staining as compared with 60.8% in those with wild-type STK11 and KEAP1 (*p* < 0.001). Patients with concurrent STK11 and KEAP1 mutations had shorter real-world mPFS when treated with PD-1/PDL-1 inhibitors, anti-VEGF, EGFR inhibitors, platinum doublets or single-agent chemotherapy. Specifically, for the patients treated with anti-PD-1/PD-L1, having co-mutation of KEAP1 and STK11 had poorer mPFS compared with mutations in either KEAP1 or STK11. Thus, co-mutation of STK11 and KEAP1 is a predictive factor for any systemic therapy, including anti-PD-1/PDL-1 therapy.

Arbour et al. examined the impact of STK11 and/or KEAP1 mutation on benefit to immunotherapy in 177 KRAS-mutant NSCLC patients [27]. The presence of KEAP1 co-mutation was found to have shorter mOS (6 months vs. not reached (NR), *p* = 0.006), while co-mutation with STK11 did not have an impact on mOS (11 months vs. NR, *p* = 0.3). Thus, KEAP1 was a predictive factor in metastatic KRAS-mutated NSCLC, treated with immunotherapy.

In the study by Skoulidis et al., metastatic adenocarcinoma NSCLC patients with KRAS mutation alone and co-mutation in STK11 were resistant to PD-1 inhibitors alone (ORR = 7.4% vs. 28.6%, *p* < 0.0001, mPFS 1.9 months vs. 2.7 months, *p* < 0.001 and mOS 6.4 months vs. 16 months, *p* = 0.0015, respectively) [28,29]. The mPFS for patients with KRAS and STK11/LKB1 co-mutation and treated with chemo-immunotherapy was 4.8 months vs. 6.9 months in those with KRAS mutation alone (HR = 1.58, 95% CI 1.20–2.08, *p* = 0.0012). In the chemotherapy arm, the mPFS for the STK11 mutant was also inferior (3.7 months versus 5.6 months, HR = 1.29, 95% CI (1.00–1.65), *p* = 0.052). The authors also reported the negative impact of STK11 mutation on the KRAS mutation patients enrolled in the CheckMate 057 trial, with an ORR of 0% vs. 18.2%, respectively [5]. However, the sample size was small, with *n* = 6 for those who did have the STK11/LKB1 mutation, and therefore, it was hard to draw a strong conclusion regarding the predictive value of this marker in immunotherapy treatments from this trial.

In summary, STK11 and/or KEAP1 mutation in KRAS-mutant NSCLC is a negative predictive and prognostic factor for immunotherapy.

## 6. Host Microbiome

Another factor that may influence immunotherapy efficacy is the gut microbiome. This refers to the intestinal microbiota, which influences the immune response and can be altered by antibiotics or other medications such as proton pump inhibitors (PPI). Several studies have shown that germ-free mice have different response to PD1/PDL-1 inhibitor and anti-CTLA4 compared with mice treated with antibiotics. Antibiotics alter the anaerobic bacteria subtypes located in the small intestine and colon. This suggests that the dysregulation of the gut microbiome influences the ability to mount an immune response in the presence of ICIs. A study by Routy et al. showed an important relationship between antibiotics affecting gut microbiota and the resulting implication for ICIs [30]. The faecal microbiota from patients responding to ICIs was transplanted into germ-free vs. antibiotic-treated mice. The faecal microbiota transplantation improved the antitumour effects of PD-1 blockade. In comparison, non-responding patients, the faecal microbiota of whom was transplanted, did not have a response to PD-1 blockade. Additionally, patients who were responders to ICIs had a higher amount of *Akkermansia muciniphila*. This suggests that the relative number of types of microbe has an effect on immune checkpoint inhibitor response.

**Figure 3:**
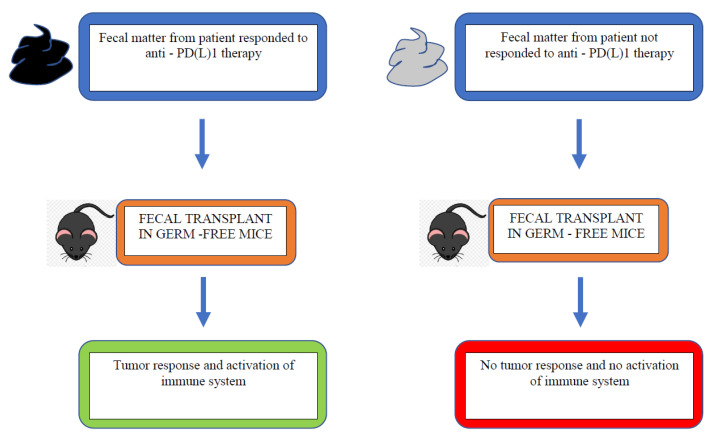
Faecal microbiota transplantation with responder vs. non-responder microbiota [30].

In the retrospective analysis of phase III OAK trials and a phase II POPLAR study, which pooled the data of 1512 patients, Chalabi et al. investigated the impact of antibiotics and PPI on the therapeutic outcomes of patients when treated with chemotherapy and immunotherapy in NSCLC (Figure 3) [31]. With the alkalinization of the stomach by PPI, the bacterial flora of both the stomach and the small and large intestine could be altered. The mOS was significantly shorter in the atezolizumab arm for patients who received antibiotics (8.5 months vs. 14.1 months, HR = 1.32, 95% CI 1.06–1.63, *p* = 0.01) and who used PPI (9.6 months vs. 14.5 months, HR = 1.45, 95% CI 1.20–1.75, *p* = 0.0001). The mOS was not significantly shorter in the docetaxel-treated patients after treatment with antibiotics (HR = 1.13 (95% CI 0.93–1.37) and PPI = 1.17 (95% CI 0.97–1.40), respectively). In the overall pooled population, the multivariate model showed poorer mOS with antibiotics and PPI use for HR with antibiotic or PPI use, being 1.20 (95% CI 1.04–1.39, *p* = 0.01) and 1.26 (95% CI 1.10–1.44, *p* < 0.01), respectively. Thus, antibiotic or PPI use resulted in a poorer outcome with immunotherapy, suggesting that they may serve as predictive factors.

A recent Italian real-world retrospective analysis aimed to study the impact of concurrent medications (antibiotics, PPI) on the clinical outcome in various stage 4 malignancies, including NSCLC (52.2% of total population) [32]. The disease progression was significantly higher in patients on prophylactic systemic antibiotics (HR = 1.85, 95% CI 1.23–2.78) and gastric acid suppressants (HR = 1.29, 95% CI 1.09–1.53).

In summary, the role of the gut microbiome in immunotherapy efficacy has not been studied in a prospective analysis, but has been demonstrated in the retrospective data. This is a key area because many patients with lung cancer are treated with antibiotics leading up to their diagnosis. The timing of antibiotics or PPI and their effect on immunotherapy efficacy remain to be explored. The exact mechanism is still unclear, and more studies are needed to understand how and if the microbiome influences the activity of T helper cells, thereby affecting immune checkpoint inhibitors.

## 7. Conclusions

While advancements in precision medicine have opened doors for targeted therapies, predicting the factors that determine which NSCLC patients will benefit from targeted therapies represents a considerable knowledge gap. While PD-L1 status is one predictive factor, we are only now beginning to learn about the role of other factors, such as the tumour mutational burden, smoking history or gut microbiome. In addition, specific genomic mutations can influence how likely it is that one will respond to immunotherapy. Further understanding in this area will help in stratifying those patients who will benefit from immunotherapy and guide clinical management.

## Figures and Tables

**Table 1:** Summary of HR for mOS based on PD-L1 expression.

Trial	Line of Therapy	Immunohistochemistry Antibody and Positivity	Agents	HR for mOS (95% CI) by PDL-1 (%)
CheckMate 017 (squamous)	Second	1%, 5%, 10% Dako 28-8	Nivolumab vs. docetaxel	HR = 0.62 (0.48–0.79)
CheckMate 057 (non-squamous)	Second	1%, 5%, 10% Dako 28-8	Nivolumab vs. docetaxel	HR = 0.70 (0.58–0.83)
CheckMate 017/057 (pooled)	Second		Nivolumab vs. Docdetaxel	>1%: HR = 0.61 (0.49–0.76) <1% HR = 0.76 (0.61–0.96)
KEYNOTE-010	Second	>1% Dako 22C3	Pembrolizumab 2 mg/kg and 10 mg/kg (pooled analysis) vs. docetaxel	>50% HR = 0.53 (0.42–0.66) >1% HR = 0.69 (0.60–0.80)
KEYNOTE-024	First	>50% Dako 22C3	Pembrolizumab vs. platinum-based chemotherapy	>50% HR = 0.63 (0.47–0.86)
KEYNOTE-042	First	>1% Dako 22C3	Pembrolizumab vs. platinum-based chemotherapy	>50% HR = 0.69 (0.56–0.85) 1–49% HR = 0.92 (0.77–1.10)
KEYNOTE-189	First	Dako 22C3	Pembrolizumab and platinum/pemetrexed vs. platinum/pemetrexed	>50% HR = 0.64 (0.37–1.10) 1–49% HR = 0.57 (0.36–0.90) <1% HR = 0.61 (0.38–0.98)
KEYONTE-407	First	Dako 22C3	Pembrolizumab and carboplatin/paclitaxel or nab-paclitaxel vs. carboplatin/paclitaxel or nab-paclitaxel	>50% HR 0.64 (0.37–1.10) 1–49% HR 0.57 (0.36–0.90) <1% HR 0.61 (0.38–0.98)
IMpower130	First	Ventana SP142	Atezolizumab and carboplatin/nab-paclitaxel vs. carboplatin/nab-paclitaxel	TC3/TC3 HR = 0.84 (0.51–1.39) TC1-2/IC1-2 HR = 0.70 (0.45–1.08) TC0/IC0 HR = 0.81 (0.61–1.08)
IMpower150	First	Ventana SP142	Atezolizumab/bevacizumab and carboplatin/paclitaxel vs. carboplatin/paclitaxel and bevacizumab	TC3/IC3 HR = 0.39 (0.25–0.60) TC1-2/IC1-2 HR = 0.56 (0.41–0.77) TC0/IC0 HR = 0.77 (0.61–0.99)
CheckMate 227	First	Dako 28-8	Nivolumab/ipilimumab vs. chemotherapy	>1% 17.1 months vs. 14.9 months, *p* = 0.007 <1% 17.2 months vs. 12.2 months
CheckMate 9LA	First	Dako 28-8	Nivolumab/ipilimumab and 2 cycles of platinum-based chemotherapy vs. 4 cycles of platinum-based chemotherapy	>50% HR = 0.67 (0.46–0.97) 1–49% HR = 0.70 (0.56–0.89) <1% HR = 0.67 (0.51–0.88)

Dako and Ventana SP142 are assays utilized for the immunohistochemistry; TC0 is defined as PD-L1 expression on <1% tumour cells; IC0 is defined as PD-L1 expression on <1% of tumour-infiltrating immune cells; TC3 is defined as PD-L1 expression on >50% tumour cells; IC3 is defined as PD-L1 expression on >50% tumour-infiltrating immune cells; TC1/2/3 is defined as PD-L1 expression on >1% tumour cells; TC1/2 is defined as PD-L1 expression in ≥1% and <50% of tumour cells; IC 1/2 is defined as ≥1% and <10% of tumour-infiltrating immune cells.
